# Assessing the impact of various tuberculin PPD brands on bovine tuberculosis diagnosis

**DOI:** 10.1038/s41598-024-52089-1

**Published:** 2024-03-02

**Authors:** Gustavo Echeverría, Martín J. Zumárraga, Freddy Proaño-Pérez, Francisco Barceló Blasco, Jacobus H. de Waard

**Affiliations:** 1 Instituto de Investigación en Zoonosis-CIZ, Universidad Central del Ecuador, Quito, Ecuador; 2 Facultad de Ciencias Veterinarias, Universidad de Buenos Aires, Buenos Aires, Argentina; 3 División Investigación y Desarrollo, BioGENA, Quito, Ecuador; 4 Instituto de Agrobiotecnología y Biología Molecular, IABIMO, INTA-CONICET, Buenos Aires, Argentina; 5 Facultad de Medicina Veterinaria y Zootecnia, Universidad Central del Ecuador, Quito, Ecuador; 6 One Health Research Group, Facultad de Ciencias de la Salud, Universidad de las Américas (UDLA), Quito, Ecuador

**Keywords:** Immunology, Microbiology, Diseases

## Abstract

Although several brands of tuberculin purified protein derivatives (PPDs) are available for diagnosing bovine tuberculosis (bTB), comparative studies to determine their diagnostic accuracy are infrequent. In Ecuador we compared two different PPD brands for bTB diagnosis using skin testing and measuring skin thickness increase. Additionally, we evaluated four PPD brands, including those used for skin testing, in the Bovine Tuberculosis Interferon Gamma Test (IFN-γ test) measuring IFN-γ induction in whole blood. The study included 17 naturally tuberculosis-infected PPD and IFN-γ test positive bovines. Both the field and laboratory results showed significant differences in classifying the 17 bovines as bTB positive or negative. We hypothesize that several factors, such as the genetic background of the cows, sensitization to environmental mycobacteria, *M. bovis* strains involved in the bTB infection, and the manufacturing procedures of the PPDs, could have influenced the immune reaction toward the different tuberculin PPD brands. Our study emphasizes the necessity for comparative studies aimed at determining the diagnostic accuracy of PPD brands for bTB diagnosis as well as the development of standardized methods for PPD production and potency determination.

## Introduction

Bovine tuberculosis (bTB) is a chronic bacterial disease caused by a *Mycobacterium bovis* infection, that affects cattle and other mammals, and it is a mandatory reportable disease to the World Organization for Animal Health (OIE)^1,2^. The disease is widely distributed worldwide and has a significant economic impact on the livestock production sector^3^. Economic losses are estimated to absorb 10–15% of livestock production due to commercial restrictions, reduced milk and meat production, costs of control programs, and culling of animals^4,5^. Additionally, bTB is considered a potential risk to human health due to its zoonotic potential^6^. Recent reports indicate that *M. bovis* accounts for 1–8% of tuberculosis (TB) cases in developed countries, while the prevalence of *M. bovis* infection remains unknown in most developing countries^6,7^.

A review study aimed at estimating the prevalence of bovine tuberculosis (bTB) in South America found that Ecuador has a relatively high prevalence of more than 1%^8^. This finding is supported by other reports from Ecuador that registered a prevalence range of 0.71–8.63%, depending on the geographic region or the methodology used such as Tuberculin skin test surveys or inspection at slaughterhouses^9,10^. In terms of the zoonotic aspect of bTB, *M. bovis* is estimated to account for 2% of all human cases of TB in South America^11^.

To diagnose bTB in cattle, the single intradermal tuberculin (SIT) test is currently the primary diagnostic tool used in most settings. This test involves injecting bovine PPD into the skin of the mid-neck or caudal fold and measuring any subsequent swelling at the injection site 72 h later^1,2,12^. An increase of 4 mm or more in skin thickness is considered a positive reaction, and the animal is typically culled. However, in some countries such as the United Kingdom and Ireland, the single intradermal comparative cervical tuberculin (SICCT) test is used. This test simultaneously injects both bovine and avian PPD into the skin of the mid-neck, and the response to the injected bovine PPD is compared to the avian PPD response 72 h post-injection. An animal is deemed positive if the bovine reaction exceeds the avian reaction by 4 mm or more. This test is believed to better distinguish between animals infected with *M. bovis* and those previously exposed to or infected with other types of mycobacteria found in the environment and that do not cause bTB^13,14^.

In recent years, the IFN-γ test has gained increasing popularity as a supplementary diagnostic tool for bTB due to its potential advantages over the SIT and SICCT tests. This test, based on the detection of IFN-γ produced by lymphocytes in whole blood samples after stimulating with avian and bovine PPD, has been extensively reviewed in several publications^15–20^ and has been shown to have higher sensitivity and specificity compared to the SIT and SICCT tests^21–24^. Two commercially IFN-γ tests are available (the ID Screen^®^ Ruminant IFN-g test by IDvet and the Bovigam^®^ test by ThermoFisher Scientific). These tests come with their own avian and bovine PPD preparations for the stimulation of the lymphocytes. However, in some countries the same avian and bovine PPD, used for field testing, is also employed for stimulation in the IFN-γ test^23,24^.

Worldwide, there is a wide range of commercial preparations or brands of avian and bovine PPD available for bTB skin testing. The World Organization of Animal Health regulates and standardizes the production of this biological, requiring it to be bio-assayed for potency determination. For cattle, bovine PPD is considered of acceptable potency if its estimated potency is at least 20,000 IU/ml (± 25%)^1,2^, and avian PPD with a potency of 25,000 IU/ml, a standard supported by field trials^25^. Higher doses of bovine PPD, up to 50,000 IU/ml, have been recommended for national eradication campaigns or cases where cattle have diminished allergic sensitivity^26–29^.

To assess the diagnostic accuracy of different PPD brands, only a few direct comparative studies have been conducted^26,27^. In the present study, our objective was to investigate the effect of using PPDs from various brands and different countries on the diagnosis of bTB. We selected a sample of 17 naturally infected cattle and conducted skin tests using two different brands of avian and bovine PPD. Additionally, we used four PPD brands, including the two brands used in the skin tests, to stimulate whole blood and measure IFN-γ levels. We then compared the skin test results, IFN-γ induction, and reactor status (positive or negative for bTB) among the different PPD brands and tests.

## Materials and methods

### Avian and Bovine tuberculin PPD

Table 1 displays information regarding the avian and bovine tuberculin PPDs used in this study and their potencies, as provided by the manufacturers. The pairs of avian and bovine PPDs were obtained from four different manufacturers located in Europe and Latin America. To maintain anonymity regarding their origin, the brands of tuberculin PPD were randomly assigned a capital letter (A, B, C, and D). Brand A and B were used in skin testing. Al four brands, diluted 1:10 in sterile PBS, were used for stimulation in the IFN-γ test.

**Table 1:** The avian and bovine tuberculin Purified Protein Derivatives (PPDs) used in this study with their respective potency as mentioned on the label**.**

Tuberculin PPD	Potency (IU/ml) as indicated by the manufacturer	Final concentration in gamma interferon test	Country of origin
Bovine	30,000	325 IU/ml	A
Avian	25,000	250 IU/ml	
Bovine	25,000	250 IU/ml	B
Avian	25,000	250 IU/ml	
Bovine	50,000	500 IU/ml	C
Avian	25,000	250 IU/ml	
Bovine	30,000	300 IU/mL	D
Avian	25,000	250 IU/ml	

### Bovines naturally infected with tuberculosis

For this study we selected a group of 17 cows from a dairy farm with approximately 650 cows of the Holstein Friesian breed, located in the Andean region of the province of Pichincha. The farm is situated at an altitude of approximately 3000 m above sea level. The farm had a confirmed history of bTB. and Samples from scarified cows have been cultured and tested positive for *M. bovis* infection on three separate occasions. Using the region of deletion method this molecular method showed that the RD4 region, which encompasses Rv1506c–Rv1516c of *M. tuberculosis*, was absent in the *Mycobacterium tuberculosis* complex strains isolated from the scarified cows. The 17 cows were selected from 83 cows that resulted positive for the single intradermal tuberculin test (SIT), with reaction between 4 and 38 mm. The selected cows were between 4 and 8 years old and all had a swelling of more than 10 mm at the injection site in the caudal fold of the tail 72 h after the single intradermal tuberculin test (SIT) using bovine tuberculin PPD brand B. In addition, these 17 cows showed a strong positive result for a tuberculosis infection, with the ID Screen^®^ Ruminant IFN-γ test (IDvet Grabels, France) with whole blood stimulated with avian and bovine PPDs (PPDPACK IDvet, France).

### Re-testing in the field with two brands of tuberculin

The 17 selected bovines underwent retesting using the single intradermal comparative tuberculin test (SICTT) with avian and bovine tuberculin PPDs from brands A and B, 11 weeks after the first SIT test to avoid desensitization of the cows^30,31^. The test was performed on both sides of the mid-neck. The skin on each side was shaved before injecting the PPD combination with a 10–12 cm separation. We measured the skin thickness using a mechanical caliper with a return spring for standard pressure, both before and 72 h after the injection. To ensure consistency, the same person performed all measurements.

### The interferon gamma test

After recording the results of the SICCT skin test, blood samples were drawn from the tail vein of the 17 tuberculous cows and collected into 4 mL vacutainer tubes containing lithium heparin. The IDvet IFN-γ tests was performed according to the manufacturer instructions. The avian and bovine PPDs that are included with the test kit (PPDPACK IDvet, France) were used as controls. Also, to ensure the blood was in good condition to assess immune function in the assay, a positive activation control for the 17 blood samples was included using mitogen (pokeweed IDvet, France). Within 6 h of collection, the blood samples were divided into twelve (12) aliquots of 250 μL and incubated with 25 μL PBS (blank), 25 μL of each of the 4 bovine PPDs (activated sample) or 25 μL of the 4 avian PPDs (control sample), 25 μL of each of the avian and bovine PPDPACK-PPDs and 25 μL mitogen. Before the blood was mixed with the different brands of bovine and avian PPDs, these were diluted 1:10 in sterile PBS. The blood was incubated at 37 °C for 20 h and plasma was collected and tested for the presence of gamma-interferon using an ELISA (ID Screen^®^ Ruminant IFN-γ test, IDvet Grabels, France) following the manufacturer's instructions. OD measurements of the ELISA assay were then transformed into sample-to-positive ratios or S/P values with the following formula: [(OD bovine PPD − OD avian PPD)/(mean OD positive control of the kit − mean OD negative control of the kit)] × 100%. The results were interpreted according to the test instructions, with stimulated samples considered positive for bTB if they had an S/P value of > 35%.

### Data analysis

Means, coefficients of variation and standard deviations were calculated with Microsoft Excel for Windows. All data generated or analyzed during the current study are included in this published article and its supplementary information files.

### Animal welfare and ethical considerations

As of now, there is no bioethical committee for animals in Ecuador. The cows used in this study were selected based on their infection with bTB by the National Bovine Tuberculosis Program, which adheres to stringent ethical guidelines and requires the cows to be euthanized in a slaughterhouse as per the program's regulations. A licensed veterinarian performed the skin test and collected blood samples from the cows a week before their transportation to the slaughterhouse. The study is reported in accordance with ARRIVE guidelines. All experimental protocols were approved by a committee of the International Center of Zoonosis, Universidad Central del Ecuador, Quito, Ecuador and all methods were carried out in accordance with relevant guidelines and regulations.

## Results

### Tuberculin PPD skin retesting

The retesting of the 17 PPD-positive tuberculous cows with PPD revealed a significant difference in skin-thickness increase between brands A and B, as shown in Table 2. Bovine PPD of brand B, which was used to select the cows as "positive" for bTB, elicited significantly superior reactions compared to brand A. The mean increase in skin thickness was 17.8 mm (SD = 7.9) for brand B and 8.2 mm (SD =1.5) for brand A. Similar results were observed for the avian PPDs, where brand B also produced superior reactions with a mean increase in skin thickness of 9.2 mm (SD = 1.4) compared to 4.3 mm (SD = 5.1) for brand A. See Table 2. All cows were classified as positive for bTB when considering the results of the SIT test with bovine PPD A or B alone since all cows had a skin thickness increase of at least 4 mm. However, there was a significant difference in the number of reactors classified as bTB positive when using comparative testing: 12/17 versus 14/17 positive reactors for the PPD sets A and B, respectively. Only 9 out of 17 reactors were classified as positive for bTB with both PPD brands, and we found a negative degree of agreement (weighted kappa − 0.28) when assessing the agreement between these classifications using brands A or B.

**Table 2:** Results of the single intradermal comparative tuberculin test (SICTT) using two different brands of PPD (A and B) in 17 bovines with tuberculosis.

Animal	Tuberculin A			Tuberculin B		
	Bovine PPD	Avian PPD	PPD(b-a) (mm)	Bovine PPD	Avian PPD	PPD(b-a)
1	8	3	5 (P)	14	9	5 (P)
2	7	2	5(P)	12	9	3 (N)
3	7	4	3 (N)	22	8	14 (P)
4	11	1	10 (P)	11	10	1 (N)
5	7	4	3 (N)	13	8	5 (P)
6	9	4	5 (P)	36	8	28 (P)
7	11	6	5 (P)	24	9	15 (P)
8	9	0	9 (P)	17	9	8 (P)
9	9	2	7 (P)	12	12	0 (N)
10	6	7	− 1 (N)	17	8	9 (P)
11	9	4	5 (P)	17	12	5 (P)
12	7	3	4 (P)	14	10	4 (P)
13	6	3	3 (N)	12	8	4 (P)
14	9	23	− 14 (N)	16	11	5 (P)
15	8	2	6 (P)	12	8	4 (P)
16	9	3	6 (P)	37	9	28(P)
17	7	2	5 (P)	17	8	9 (P)
Mean increase (mm) and SD	8.2 SD 1.5	4.3 SD 4.9	Total positives 12/17	17.8 SD 7.6	9.2 SD 1.3	Total positives 14/17

Each cow was injected with bovine and avian PPD from brand A on the right side of the mid-neck and bovine and avian PPD from brand B on the left side. The skin thickness increase was measured to the nearest 0.1 mm, but intermediate results were rounded to the nearest whole number. A positive test result is indicated by a "P" and was calculated as a relative increase in skin thickness of 4 mm or more for bovine PPD minus the increase for avian PPD. Negative test results are indicated with "N".

### The bovine tuberculosis interferon-gamma assay

**Figure 1:**
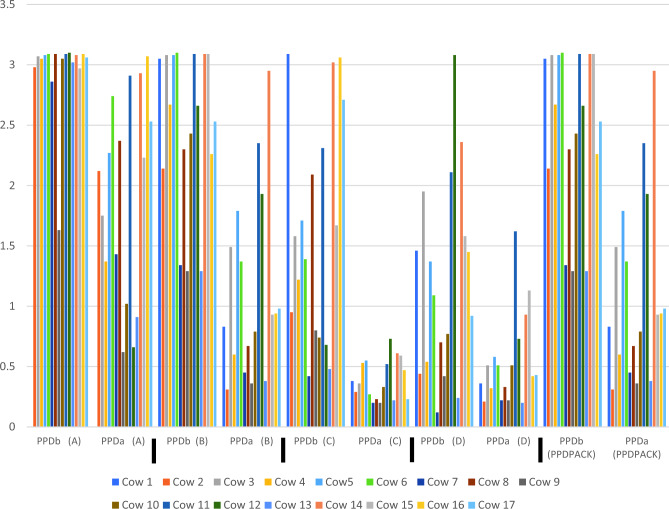
Optical densities of the ELISA readings (relative amount of induced INF-y in plasma) after stimulating heparinized whole blood of 17 Tuberculosis cows with bovine and avian PPD of 4 tuberculin brands (**A**–**D**) and PPDPACK (IDvet). OD readings were corrected for the OD of plasma without stimulation. *PPDb*=bovine PPD, *PPDa*=avian PPD.

Five pairs of bovine and avian PPDs were available for the IFN-γ assay; brands A and B, which were used in the skin testing, as well as brands C, D, and the brand provided in the IDvet ELISA kit. To ensure the blood samples were in good condition for immune assessment, pokeweed mitogen (IDvet, France) was used as a positive activation control. All 17 blood samples stimulated with pokeweed showed a S/P value of > 250% after 20 h of stimulation (results not shown). Also, all blood samples tested positive for bTB with S/P values of > 35% (mean S/P value = 188%) after stimulation with the avian and bovine PPDs included in the IDvet ELISA kit (see Table 3). However, the results of the stimulation with the PPD brands A-D were significantly different and respectively three, one, three, and five cows out of 17 were tested as negative. Only 7/17 cows were classified as bTB positive with the paired tuberculin PPDs from all four brands (Table 3). These results suggest that the brand of PPD affects the test results of the IFN-γ assay. Furthermore, there was no correlation between the increase in skin thickness induced by brand A and B and the optical density (OD) in the IFN-γ assay. Brand A induced the highest OD readings in the IFN-γ assay, respectively 20% and 60% more for bovine PPD and avian PPD than brand B but induced a lower increase in skin thickness in the tuberculosis skin test compared to brand B (Table 2). Additionally, the OD values in the ELISA reading for samples stimulated with bovine PPD with a registered potency of 50,000 IU/mL were with an average of about 40% lower than the OD reading after stimulation with bovine PPD of brand A (see Fig. 1; Supplementary File 1). Moreover, most of the 17 animals had a strong response to bovine PPD of most of the brands; however, the responses to the avian PPDs were very variable. A stacked bar chart displaying the cumulative positive test results of individual animals can be found in Supplementary File 2. We used a statistical method called Gwet's AC_1 coefficient to see how much agreement there was between the results of the different tests. The estimated agreement between the different SICTT tests was calculated as 0.38 with a standard error of 0.24 and a p value of 0.13, meaning a chance-corrected agreement not significantly distinct of zero. Between the INF-γ results the estimated agreement was 0.69 with a standard error of 0.13 and a p value of 0,008 meaning a positive agreement between the tests.

**Table 3:** Interpretation of the results of Gamma Interferon testing of 17 tuberculous cows, using whole blood stimulated with four different brands of tuberculin PPD (brand A, B, C, and D).

Animal	PPD A	PPD B	PPD C	PPD D	PPDPACK
1	ND*	P	P	P	P
2	P	P	P	P	P
3	P	P	P	P	P
4	P	P	P	Negative	P
5	P	P	P	P	P
6	P	P	P	P	P
7	P	P	Negative	Negative	P
8	P	P	P	P	P
9	P	P	P	Negative	P
10	P	P	P	Negative	P
11	Negative	P	P	P	P
12	P	P	Negative	P	P
13	P	P	Negative	Negative	P
14	Negative	Negative	P	P	P
15	P	P	P	P	P
16	Negative	P	P	P	P
17	P	P	P	P	P
Positive	13/16	16/17	14/17	12/17	17/17

As a control, PPDPACK, the avian and bovine PPD recommended by the manufacturer (IDvet, France) for in vitro cell activations was used. The OD readings of the ELISA after stimulation with PPD-b and PPD-a can be found in Supplementary Figs. 1 and 2. A cow was considered positive for the INF-γ test if the S/P value of the ELISA readings was more than 30%, while an S/P value < 30% was considered negative for the test. See also the Supplementary files 1 and 2 for the OD readings and S/P values of the ELISA.

*For this cow, no blood was available for testing with the PPD of manufacturer A.

## Discussion

Our study, which was conducted in Ecuador, South America, and aimed to evaluate the performance of four pairs of bovine and avian PPDs from different brands using both skin testing and the IFN-γ assay. We utilized two brands in skin testing and four brands, including the ones used in skin testing, in the IFN-γ test. Our findings indicated that the brand of PPD significantly influences the test results, resulting in varying responses observed in both the skin test and IFN-γ assay in 17 tuberculous cattle. These variations could potentially lead to missed diagnoses or false negatives, ultimately impacting bTB control and eradication programs.

### Comparison of PPD activity in skin testing

We found that brand B induced a significantly higher increase in skin thickness than brand A for both avian and bovine PPD. However, this difference did not affect the final interpretation of the comparative intradermal tuberculin (CIT) test, which relied on the results of bovine PPD only. All 17 cows should have been classified as reactors based on the CIT criteria of a 4 mm or greater increase in skin thickness.

On the other hand, the high variability of skin reactions to avian PPD had a significant impact on the final results of the single intradermal comparative cervical tuberculin (SICCT) test, leading to poor agreement between testing with bovine PPD from brand A or B. Notably, the differences in skin thickness increase between the brands were not related to the reported potency of the PPDs, as the PPD with the highest potency (PPD A) caused the lowest increase in skin thickness.

One possible explanation for the difference in skin thickness increase between the brands is that brand A may have been partially inactivated during transport to our country. However, this seems unlikely given previous studies showing the high stability of PPD even under extreme conditions^32,33^. Another possibility is that previous skin testing with brand B, used to select the bovines in our study, may have affected the subsequent skin test and primed these animals for the B brand PPD. However, there is no support for this hypothesis in the literature. Moreover, it is generally believed that the inoculation of antigens from mycobacteria in the skin negatively affects subsequent reactivity to the same antigens in naturally infected animals, and desensitization has been reported after repeated exposure to mycobacterial antigens^34,35^. Only a limited number of other studies have compared different brands of PPDs in the same herd and same animal and these studies do not support our findings. A study in Great Britain suggested slightly higher test sensitivity and lower test specificity associated with Weybridge tuberculin compared with Lelystad tuberculin^32^. Another study in Ireland^27^ compared the performance of a number of different tuberculin combinations (that is, pairings of bovine and avian PPD; with different manufacturers) in the single intradermal comparative tuberculin test (SICTT). This study showed that the measured skin thickness differences were minor.

### Comparison of PPD activity using the INF-γ test

The ELISA-OD values, and thus INF-γ stimulation, were significantly higher in blood samples stimulated with PPD A or B brands compared to PPD C or D brands. Likewise, the results of these tests also exhibited variability in the classification of reactor status among the 17 animals. Only 8 out of 17 cows were identified as positive using all four PPD combinations. Furthermore, no correlation was found between IFN-γ production and skin thickness swelling for brands A and B. Although PPD brand B produced significantly greater swelling, it induced lower levels of INF-y. Additionally, the bovine PPD of brand C, the brand with the highest reported potency, did not produce the highest INF-y levels. Concerning the literature, in one study only minor differences in INF-y responses to two commercial PPDs were observed with samples from experimentally infected animals^36^. However, another study indicated significant differences between PPDs from various sources and emphasized the need for standardization of PPDs using in the IFN-γ assay^37^.

### Production of PPD

Another possible explanation for the observed differences in immunogenicity between the brands of PPD could be the differences in their manufacturing methods. Although all PPDs are supposed to be produced according to World Organization for Animal Health (OIE) standards and prepared from the culture filtrate of *M. bovis* strain AN5 and *M. avium* strain D4^26,27^, no specific culture medium has been indicated in the standards^1,38^. Therefore, different synthetic media, such as Long's medium, Sauton medium, modified Reid’s medium or Dorset–Henley medium, can be used for their preparation. These differences in the type of medium used for the production can lead to different proteins being expressed in the different brands of PPDs. Few studies have been undertaken to compare culture medium and PPD quality, but one interesting study compared Sauton medium and its modification with double the amount of asparagine for the production of PPD. The modified medium resulted in a considerably greater PPD yield, and the PPD from the modified medium was not less potent per unit of weight than that from the unmodified medium, although there were differences in the proportions of the different protein components^39,40^.

To expand on the potential differences between PPD preparations, it's important to note that the method used for their protein precipitation can also vary. Two common methods are ammonium sulfate (AS) and trichloroacetic acid (TCA) precipitation. While TCA precipitation has been shown to deliver a higher protein recovery compared to ammonium sulfate, it may partially denature the proteins, which could affect the stimulation of T cells^41^. Additionally, the SDS-PAGE pattern of proteins precipitated with TCA showed proteins with a wide range of molecular weight, while proteins precipitated with AS showed predominantly low molecular weight proteins^42^. These differences in protein precipitation methods could lead to varying protein contents in PPDs, which in turn may affect their immunogenicity and performance in skin tests or blood stimulation immunogenic reagent. Therefore, it's important to consider both the culture medium and protein precipitation method used when comparing PPD preparations.

The variation in protein content among PPDs from different manufacturers has also been substantiated through proteomic studies. Several articles that have investigated the protein composition of various PPD preparations that are employed in the control and surveillance of bTB and report distinct proteomic profiles^43–45^. These findings underscore the fact that no two PPDs are identical. While PPDs may share certain proteins, it is important to note that a significant portion of their total protein content, up to 25%, is exclusive to certain PPDs and absent in others^45^.

### Other factors that could have affected the immunoreactivity of the PPDs

Various other factors such as the genetic background of the cattle, environmental sensitizations of the cows with nontuberculous mycobacteria and/or the *M. bovis* strains involved in the infection of our cattle could also have played a significant role in the sensitivity and specificity of the tested PPDs. For instance, a study by Aagaard et al. found large site-to-site variations in the sensitivity of specific immunodominant antigens with the INF-γ test among skin test positive cattle in three different settings; in Northern Ireland, Argentina, and Mexico^46^. The authors concluded that the genetic background of the cows, environmental sensitizations, and the *M. bovis* strains involved in the infection may have played significant roles in the immunoreactivity of these proteins. Similarly, studies in humans have shown that genetically different *M. tuberculosis* genotypes can evoke markedly different immune responses in the host^47^. It has also been shown that the PPD reactivity of infected humans depends on their genetic background^48^. These findings highlight the complex interplay between the host, pathogen, and the host immune response in determining the immunogenicity of PPDs.

## Conclusions

Tuberculin purified protein derivatives (PPDs) are widely used for screening cattle as an aid in the diagnosis of tuberculosis, despite their crude nature and poorly defined active components^49,50^. Although international organizations such as the European Pharmacopeia, WHO, OIE, and EU have established standards for PPD^1,2^, there is no independent body or study that has evaluated commercially available PPD preparations and determined variation in potency between batches or brands, or in cattle herds with different genetic backgrounds or from different geographical and climate areas. As demonstrated by our study, the variations in the performance of tuberculin may be due to differences in production methods between brands or between different batches from a given manufacturer^26,27,51^. Therefore, it is crucial to conduct independent evaluations of commercially available PPDs and to undertake further research to improve the diagnostic accuracy of bTB testing in diverse settings.

### Limitations of this study

The seventeen cows came from a farm with a confirmed prevalence of approximately 20% of bTB and were considered tuberculous due to a strong reaction to bovine PPD brand B (greater than 10 mm) and a high positive S/P value of 188 (SD 67) in the IFN-γ assay with the PPDPACK avian and bovine PPD. However, the animals were not confirmed to have bTB at the slaughterhouse during post mortem inspection. Post-mortem inspection is usually not done in our country as it is known that in general the visible lesion detection rate is generally low and highly variable^52–55^. Concerning the IFN-γ assay, this test was performed only once in this study due to the relatively high costs of this kit thus no data is available to determine the reproducibility of the test.

### Supplementary Information

Supplementary Information 1.

Supplementary Information 2.

## Data Availability

All data generated or analyzed during the current study are included in this published article and its supplementary information files.

## Abbreviations

PPD: Tuberculin purified protein derivatives
avian PPD: *M. avium*-derived PPD
bovine PPD: *M. bovis-*derived PPD
IFN-γ: Interferon gamma bTB bovine tuberculosis
