# Supplementary material for: Assessing the impact of various tuberculin PPD brands on bovine tuberculosis diagnosis
Source: Sci Rep. 2024 Mar 2;14:5155. doi: 10.1038/s41598-024-52089-1 (PMC10908831; doi:10.1038/s41598-024-52089-1)
Supplement: Supplementary file 1 — Supplementary Information 1. [file 41598_2024_52089_MOESM1_ESM.docx]

**Supplementary file 1.** Optical densities of the ELISA readings (relative amount of induced INF-y in plasma) after stimulating heparinized whole blood of 17 Tuberculosis cows with 4 brands of bovine and avian PPD (Band A respectively) and PPDPACK (IDvet). OD readings were corrected for the OD of plasma without stimulation (nil). Cows positive for bTB (S/P value of more than 30%) are indicated in a red box. Negative for bTB is a yellow box. Mean value of the OD readings with a standard deviation (SD) was calculated for each of the PPDs and are shown in the last row

|  | **Tuberculin A** | | | **Tuberculin B** | | | **Tuberculin C** | | | **Tuberculin D** | | | **PPD Pack kit** | | |
| --- | --- | --- | --- | --- | --- | --- | --- | --- | --- | --- | --- | --- | --- | --- | --- |
| Bovine | **B** | **A** | **S/P** | **B** | **A** | **S/P** | **B** | **A** | **S/P** | **B** | **A** | **S/P** | **B** | **A** | **S/P** |
| 1 | ND | ND | ND | 3,05 | 0,83 | 257,87 | 3,09 | 0,38 | 314,35 | 1,46 | 0,36 | 127,72 | 3,06 | 1,28 | 206,39 |
| 2 | 2,98 | 2,12 | 99,48 | 2,14 | 0,31 | 212,32 | 0,95 | 0,29 | 77,63 | 0,54 | 0,21 | 36,96 | 1,92 | 0,30 | 188,15 |
| 3 | 3,07 | 1,75 | 153,40 | 3,08 | 1,49 | 183,96 | 1,58 | 0,36 | 141,08 | 1,95 | 0,51 | 166,76 | 3,08 | 1,12 | 228,59 |
| 4 | 3,05 | 1,37 | 195,47 | 2,67 | 0,60 | 241,60 | 1,22 | 0,53 | 79,95 | 0,54 | 0,32 | 25,80 | 2,84 | 0,32 | 292,62 |
| 5 | 3,08 | 2,27 | 98,35 | 3,08 | 1,79 | 149,45 | 1,71 | 0,55 | 135,04 | 1,37 | 0,58 | 92,27 | 3,08 | 0,85 | 259,27 |
| 6 | 3,09 | 2,74 | 42,03 | 3,10 | 1,37 | 200,70 | 1,39 | 0,27 | 130,39 | 1,09 | 0,51 | 67,40 | 3,10 | 0,68 | 281,23 |
| 7 | 2,86 | 1,43 | 174,10 | 1,34 | 0,45 | 103,43 | 0,42 | 0,20 | 25,57 | 0,12 | 0,22 | -11,04 | 1,08 | 0,33 | 86,69 |
| 8 | 3,09 | 2,37 | 87,35 | 2,30 | 0,67 | 190,24 | 2,09 | 0,23 | 215,92 | 0,70 | 0,33 | 42,65 | 2,63 | 0,44 | 255,32 |
| 9 | 1,63 | 0,62 | 115,80 | 1,29 | 0,36 | 106,62 | 0,80 | 0,20 | 68,74 | 0,42 | 0,22 | 22,57 | 1,59 | 0,22 | 158,20 |
| 10 | 3,05 | 1,02 | 233,91 | 2,43 | 0,79 | 189,29 | 0,74 | 0,33 | 47,21 | 0,77 | 0,51 | 29,36 | 2,23 | 0,61 | 185,95 |
| 11 | 3,09 | 2,91 | 21,11 | 3,09 | 2,35 | 85,20 | 2,31 | 0,52 | 206,45 | 2,11 | 1,62 | 56,53 | 3,09 | 2,78 | 36,04 |
| 12 | 3,10 | 0,66 | 280,85 | 2,66 | 1,93 | 84,40 | 0,68 | 0,73 | -5,76 | 3,08 | 0,73 | 271,04 | 2,13 | 0,40 | 199,19 |
| 13 | 3,02 | 0,91 | 258,89 | 1,29 | 0,38 | 104,09 | 0,48 | 0,22 | 29,82 | 0,24 | 0,20 | 4,84 | 1,38 | 0,26 | 128,84 |
| 14 | 3,08 | 2,93 | 18,33 | 3,09 | 2,95 | 16,35 | 3,02 | 0,61 | 277,49 | 2,36 | 0,93 | 165,11 | 3,09 | 2,52 | 66,09 |
| 15 | 2,97 | 2,23 | 90,41 | 3,09 | 0,93 | 248,59 | 1,67 | 0,59 | 124,47 | 1,58 | 1,13 | 51,35 | 3,10 | 0,67 | 279,33 |
| 16 | 3,09 | 3,07 | 2,32 | 2,26 | 0,94 | 151,07 | 3,06 | 0,47 | 297,64 | 1,45 | 0,42 | 118,71 | 3,07 | 1,62 | 167,76 |
| 17 | 3,06 | 2,53 | 65,24 | 2,53 | 0,98 | 189,06 | 2,71 | 0,23 | 301,34 | 0,92 | 0,43 | 58,69 | 3,07 | 1,59 | 179,47 |
| Mean and SD | 2.96  0.36 | 1.93  0.84 |  | 2.50  0.66 | 1.12  0.76 |  | 1.64  0.93 | 0.39  0.17 |  | 1.22  0,82 | 0.54  0.38 |  | 2.56  0.70 | 0.94  0.79 |  |
