# Supplementary material for: Assessing the impact of various tuberculin PPD brands on bovine tuberculosis diagnosis
Source: Sci Rep. 2024 Mar 2;14:5155. doi: 10.1038/s41598-024-52089-1 (PMC10908831; doi:10.1038/s41598-024-52089-1)
Supplement: Supplementary file 2 — Supplementary Information 2. [file 41598_2024_52089_MOESM2_ESM.docx]

**Supplementary file 2**.

A stacked bar chart displaying the cumulative positive test results of individual animals. The horizontal scale represents the 17 animals. Each of the six tests is represented by a unique color. Four animals recorded six positive results, seven bovines achieved five positive results, and three and two animals respectively attained four and three positive test results. The tests used are SICTT (Single Intradermal Comparative Cervical Tuberculin Test) with PPD sets A and B in the color light blue and orange and the IFN-γ test with the PPD sets A (grey), B (yellow), C (dark blue), and D (green) from the different manufacturers."

We used a statistical method called Gwet's AC_1 coefficient to see how much agreement there was between the different tests. This method is better than Fleiss' Kappa because it takes into account the element of chance in the raters' agreement. In our results, we found that the agreement between the different SICTT tests was 0.38, which means there was some agreement, but it wasn't significantly different from what could happen by chance. However, for the INT-γ test, the agreement was 0.69, which means there was a clear and positive agreement.
